# Growth differentiation factor-15 improves long-term mortality risk prediction beyond the GRACE 2.0 score after acute coronary syndrome

**DOI:** 10.1038/s41598-026-38905-w

**Published:** 2026-02-05

**Authors:** Joel Lenell, Bertil Lindahl, David Erlinge, Tomas Jernberg, Jonas Spaak, Tomasz Baron

**Affiliations:** 1 Department of Medical Sciences, Cardiology, Uppsala University, Uppsala, Sweden; 2 Uppsala Clinical Research Center, Uppsala University, Uppsala, Sweden; 3 Department of Clinical Sciences, Cardiology, Lund University, Skåne University Hospital, Lund, Sweden; 4 Department of Clinical Sciences, Division of Cardiovascular Medicine, Danderyd University Hospital, Karolinska Institutet, Stockholm, Sweden

**Keywords:** Acute coronary syndrome, Myocardial infarction, Ejection fraction, Strain, Mortality, Outcome, Cardiology, Acute coronary syndromes

## Abstract

This study examined whether Growth Differentiation Factor-15 (GDF-15) and echocardiographic measures of systolic left ventricular function improve intermediate- and long-term mortality risk prediction beyond the guideline-endorsed GRACE 2.0 score after Acute Coronary Syndrome (ACS). 751 ACS patients were included. GDF-15, left ventricular ejection fraction (LVEF), and global longitudinal strain (GLS) were added stepwise to GRACE 2.0 in Cox regression models. Discriminative performance was assessed using the C-index for all-cause mortality at 3 years and long-term up to a median follow-up of 6.4 years. Mean age was 64.4 years, and 77% were men. There were 40 deaths at 3 years and 104 deaths by end-of-study. GDF-15 outperformed GRACE 2.0 for 3-year mortality prediction (time-dependent AUC 0.82 [95% CI 0.75–0.89] vs. 0.76 [95% CI 0.67–0.84]; *P* = 0.001). Adding GDF-15 to GRACE 2.0 improved long-term prognostic accuracy, increasing the C-index from 0.74 (95% CI 0.69–0.79) to 0.76 (95% CI 0.70–0.81). LVEF and GLS improved the C-index in the order of 0.01 when added to GRACE 2.0. GDF-15 meaningfully improved discrimination of all-cause death, both at intermediate- and long-term follow-up, when added on top of GRACE 2.0 whereas LVEF and GLS both provided minor improvements.

**Supplementary Information:**

The online version contains supplementary material available at 10.1038/s41598-026-38905-w.

## Introduction

The Global Registry of Acute Coronary Events (GRACE) risk score is one of the most endorsed risk stratification scores in Acute Coronary Syndrome (ACS) and was initially introduced as a tool in risk stratification of ACS patients on admission to guide timing of coronary intervention^1,2^. This original version of GRACE is based on a numeric calculation with risk stratification into three risk groups based on a total score. A more recent version, GRACE 2.0, is based on the same items but estimates risk using a nonlinear regression model that gives an individual point estimate of mortality probability with improved discrimination compared to the earlier version^3^. GRACE 2.0 has previously shown to offer intermediate-term prognostic value, being validated for mortality prediction up to three years after ACS^3–5^.

However, the application of GRACE score alone in longer-term risk prediction after ACS is likely not the optimal alternative. Since the introduction of GRACE score, several highly prognostic biomarkers have emerged and perhaps one of the strongest predictors of survival in ACS patients is Growth Differentiation Factor 15 (GDF-15)^6–9^. This biomarker is part of the Transforming Growth Factor (TGF)-beta family and GDF-15 expression is induced in cardiomyocytes after a myocardial infarction, serving to protect the myocardium^10^. The recently introduced ABC-ACS ischemia risk score make use of GDF-15 and was reported to outperform GRACE 2.0 in its validation cohort^7^.

GRACE also lacks prognostic information derived from imaging measurements. Left Ventricular Ejection Fraction (LVEF) by echocardiography is a well-established metric in ACS risk prediction that has been adopted to guide both pharmacological and device treatments^1^. A novel echo measurement that is increasingly adopted in assessments of systolic LV function is Global Longitudinal Strain (GLS). GLS has already proven to be a sensitive marker of LV dysfunction particularly in hypertrophic and cardio-toxic conditions^11,12^. A deterioration in GLS often precedes a reduction in LVEF, enabling earlier detection of cardiomyopathy^13^.

In this study we hypothesized that adding GDF-15 and echo measurements, LVEF and GLS, to GRACE 2.0 would improve risk assessments of future death. This investigation will help clinicians to evaluate the risk markers that best reflect long-term survival in the ACS population.

## Methods

### Population and outcome

The study population of 1385 participants was recruited between March 2008 and September 2014 as part of the TOTAL-AMI (Tailoring Of Treatment in All comers with Acute Myocardial Infarction) cohort. TOTAL-AMI is a project using data from the SWEDEHEART registry to study implications of common comorbidities in MI subtypes. Diagnoses of MI were made in clinical practice by the treating physicians, guided by the current universal definition of MI^14,15^. Subjects were recruited on admission at three participating clinics (in Uppsala, Stockholm and Lund)^5^. Inclusion and exclusion criteria are reported in the Supplemental Material. All participants provided written informed consent in accordance with the inclusion criteria before enrollment. The primary outcome was time to all-cause death and data on survival status, including date of death, were retrieved from the National Patient Register in July 2018.

### GRACE 2.0 risk score

GRACE is calculated from eight items: age, heart rate, systolic blood pressure, creatinine, cardiac arrest at admission, ST-segment deviation, elevated levels of a marker of myocardial injury (above the 99th percentile) and Killip class (1. No signs of heart failure; 2. Rales on auscultation; 3. Pulmonary edema; 4. Cardiogenic shock). The probability of death according to GRACE 2.0 was calculated for each study participant using clinical data from admission to the cardiac intensive care unit previously reported to the SWEDEHEART registry.

### Echocardiographic assessments

All echocardiographic examinations were performed as part of clinical routine within 72 h from admission. For the purpose of this study, LVEF and GLS were re-assessed at the core lab in Uppsala by one experienced reader (first author). Subjects with incomplete examinations or insufficient image quality, as determined by the reader, were excluded. The exams were analysed using TomTec-Arena version 2.30 (TomTec, Unterschleißheim, Germany) software. Volumetric measurements were obtained by manual delineation of the endocardial border in end-systole and end-diastole. LVEF was calculated according to the biplane Simpson’s rule. Peak endocardial GLS was assessed by software-automated speckle tracking of the endocardial border in the apical four-, two- and three-chamber views. In cases without a feasible three-chamber view, GLS was averaged from the four- and two-chamber views. Cases with suboptimal tracking of the endocardium in more than two segments in a single view were excluded in accordance with European and American guidelines^16^.

### Growth differentiation factor 15

GDF-15 measurements were performed in plasma ethylenediaminetetraacetic acid samples that had been stored in aliquots at −80˚C. Analyses were done using a Proximity Extension Assay chip (PEA) (Olink Proseek^®^ Multiplex CVD I 96 × 96 chip; Olink Proteomics, Uppsala, Sweden). This technology is based on pairs of antibodies, independently binding to the target biomarker; the two antibodies carry matching DNA molecules that hybridize when in proximity of each other after binding. A DNA polymerase consequently produce a new biomarker-unique DNA sequence that is amplified and quantified by real-time polymerase chain reaction (PCR). The results are presented in relative log2-transformed normalized protein expression units. The PEA technology is well validated and its use in quantification of GDF-15 have been reported to strongly correlate with immunoassay measurements^17^.

### Statistics

Variable distribution was determined by visual assessment of frequency histograms with variables of approximately normal distribution reported as mean (standard deviation) and skewed variables reported as median (interquartile range). Outliers were included in the analyses to maintain the real-world quality of the dataset.

Univariable Cox regression models were built for GRACE 2.0, GDF-15, LVEF and GLS alongside pre-specified covariables to be adjusted for in the multivariable analyses (age, sex and heart failure treatment).

To test the primary hypothesis of improved mortality prediction from the addition of GDF-15, LVEF and GLS to GRACE 2.0, each variable was step-wise added individually to a baseline model of GRACE 2.0 in nested multivariable Cox regression analyses. Following the addition of each individual variable to the baseline model, the echocardiographic metrics were step-wise added to a model with both GRACE 2.0 and GDF-15. LVEF and GLS were not analysed together in the same model due to their known moderate correlation. Harrell’s C-index was determined for each nested Cox regression model to evaluate improvement in discrimination from the variables’ joint predicted probability of time to all-cause death and change in model fit was tested with the likelihood ratio test.

Only linear terms were fitted in the main analyses to avoid overfitting, however, discriminative performance for both linear and cubic spline curves is reported in the Supplements using C-index, bootstrap-corrected C-index and calibration slope for model evaluation, together with partial effect plots.

LVEF is used to direct medical management in clinical practice and protective effects of medication may therefore diminish its prognostic impact in non-adjusted models. This was accounted for by including treatment with RAAS-inhibitors and/or beta-blockers in all multivariable Cox regression models. All multivariable models were also adjusted for age and sex as pre-specified co-variables. Age was included despite also being part of the GRACE 2.0 risk score because of weights applied to the age variable in calculation of GRACE 2.0, effectively altering its linearity.

The proportional hazards assumption for Cox regression analyses was investigated by visual inspection of Schoenfeld residual plots for each covariable. Probability of death according to GRACE 2.0 presented a non-random pattern with a predominance above zero in the Schoenfeld residual plot which was corrected by log-transformation of this variable. Given the log-transformation of GRACE 2.0 and GDF-15, hazard ratios for these variables were reported per tertile increase for easier interpretation.

All Cox regression analyses were performed with the endpoints all-cause death at three years follow-up from inclusion, given that GRACE 2.0 has been validated for this follow-up time, and at the end-of-study with a median follow-up of 6.4 years. Because of the relatively low number of events at three years, we performed internal validation using bootstrap resampling (1000 iterations) with reporting of shrunk hazard ratios alongside original hazard ratios to assess model stability.

In exploratory analyses, the individual discriminatory performance of LVEF, GLS and GDF-15 in relation to GRACE 2.0 was compared using time-dependent AUC with right-censoring at 3, 6 and 9 years^18,19^.

The C-index for GDF-15, GRACE 2.0, and the combination of the two, was plotted in a figure at 3, 6 and 9 years with the time-dependent Brier score for each model reported in an accompanying table. The prognostic difference between the tertiles of GDF-15 and GRACE 2.0 were assessed in additional exploratory analyses and depicted in Kaplan-Meier curves for each variable.

Statistical testing was performed in SPSS version 26.0 (IBM Corp. Released 2019. IBM SPSS Statistics for Windows, Version 26.0. Armonk, NY: IBM Corp) and R (4.0.2). All hypotheses were two-sided and level of statistical significance was set to *P* < 0.05.

This study was performed in line with the principles of the Declaration of Helsinki and the Global Code of Conduct. Approval was granted by the Regional Committee for Medical Research Ethics (DNR 2017/759 − 31).

## Results

### Population characteristics

**Fig. 1:**
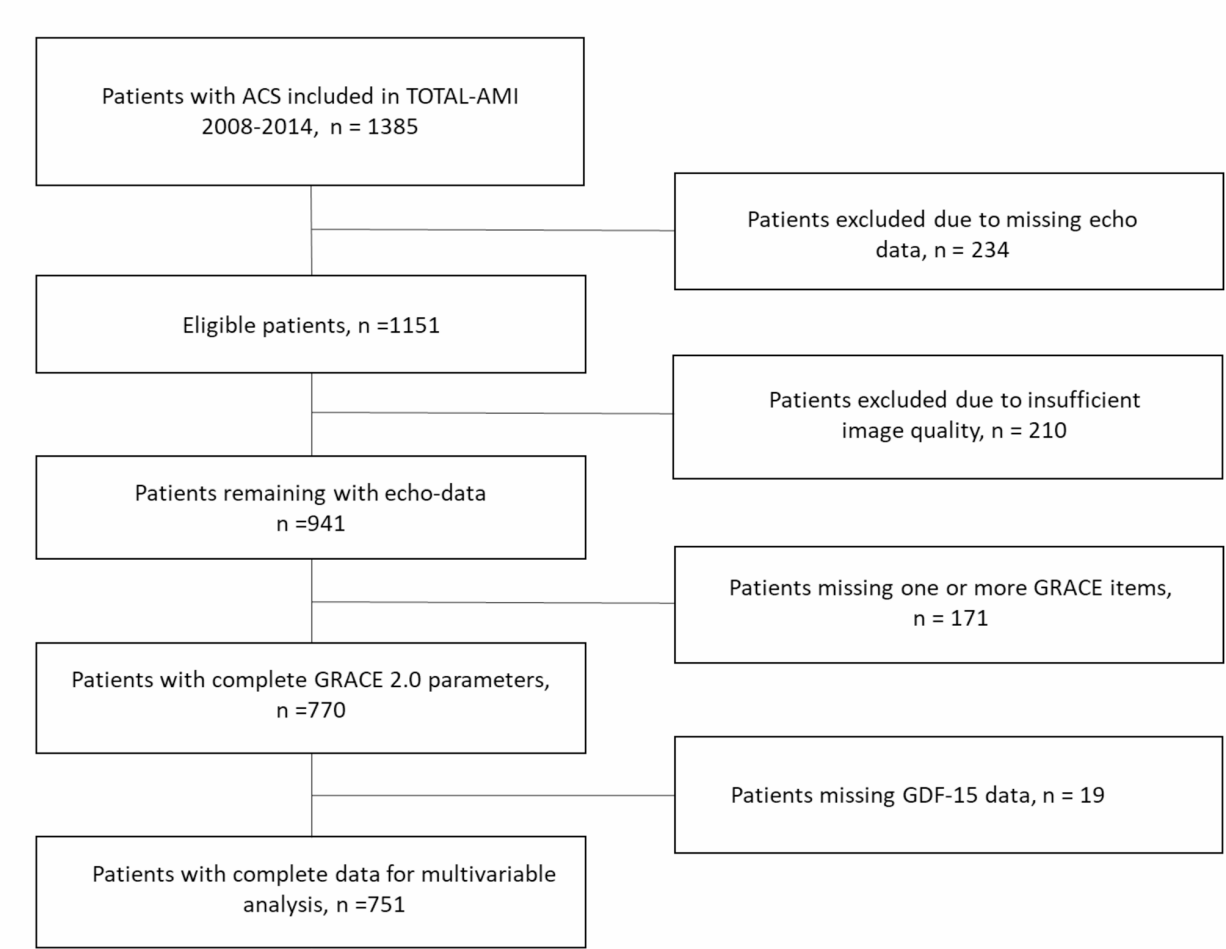
Inclusion chart. After exclusion of subjects without readable echocardiographic examinations and complete GRACE 2.0 data, 751 patients remained for analysis.

Among the 751 patients included (Fig. 1), the mean age was 64.4 years, 77% were men and 45.9% suffered a STEMI (Table 1). Percutaneous Coronary Intervention (PCI) was performed in 82.7% of patients. Median LVEF was 55% (interquartile range [IQR] 47–60) and median GLS was − 15.1% (IQR − 18.0 to −11.9).

**Table 1:** Baseline characteristics.

	*n* = 751
Male sex, *n* (%)	583 (77.6)
Diagnosis of ACS	
STEMI, n (%)	345 (45.9)
NSTEMI, n (%)	350 (46.6)
Unstable angina pectoris, n (%)	31 (4.1)
Unspecified ACS, n (%)	25 (3.3)
Revascularization	
Angiography, n (%)	728 (96.9)
PCI, n (%)	621 (82.7)
CABG, n (%)	27 (3.6)
Treatment at discharge	
Aspirin, n (%)	728 (96.9)
P2Y12-inhibitor, n (%)	668 (88.9)
Beta-blocker, n (%)	681 (90.7)
Statin, n (%)	724 (96.4)
Oral anticoagulant, n (%)	67 (8.9)
RAAS-inhibitor, n (%)	630 (83.9)
GRACE items	
Age, years, mean (SD)	64.4 (10.0)
Heart rate, mean (SD)	77.2 (19.4)
Systolic blood pressure, mean (SD)	148.8 (27.2)
Creatinine (umol/L), mean (SD)	87.5 (41.2)
Cardiac arrest at admission, n (%)	9 (1.2)
ST segment deviation, n (%)	451 (60.1)
Abnormal cardiac Troponin, n (%)	538 (71.6)
Killip class, n (%)	
1.	706 (94.0)
2.	38 (5.1)
3.	4 (0.5)
4.	3 (0.4)
Medical history	
Hypertension, n (%)	386 (51.4)
Diabetes mellitus, n (%)	163 (21.7)
Heart failure, n (%)	39 (5.2)
History of stroke^a^, n (%)	49 (6.5)
History of myocardial infarction^a^, n (%)	138 (18.4)
Chronic kidney disease (eGFR < 60mL/min/1.73m^2^), n (%)	112 (14.9)
Echocardiographic parameters	
LVEF, median (inter quartile range)	55 (47 to 60)
LV GLS, median (inter quartile range)	−15.1 (−18.0 to −11.9)

*CABG* coronary artery by-pass graft surgery, *RAAS* renin-angiotensin-aldosterone system, *GLS* global longitudinal strain, *IQR* inter quartile range, *LVEF* left ventricular ejection fraction, *NSTEMI *non-ST-segment elevation myocardial infarction, *PCI* percutaneous coronary intervention, *SD* standard deviation, *STEMI* ST-segment elevation myocardial infarction.

Included patients more often had pre-existing hypertension compared to excluded patients whereas excluded patients had a higher prevalence of renal failure and higher GDF-15 levels (Supplemental Table 1).

At three years, 40 patients had died and by the end-of-study (median 6.4 years) 104 patients had died. Non-survivors were older, had a greater burden of comorbidities at baseline and had worse LV systolic function as measured by both LVEF and GLS (Supplemental Table 2).

### Outcome analysis

Univariable Cox regression models are reported in Supplemental Table 3 for time to death up to three years and in Supplemental Table 4 for time to death up to the final follow-up. In these unadjusted analyses, GRACE 2.0, GDF-15, LVEF, GLS, age and heart failure treatment all emerged statistically significant whereas male sex was not associated with all-cause death.

Multivariable analyses for all-cause death at three years are presented Table 2. In this analysis, GRACE 2.0 was not statistically significant when adjusted for baseline age, sex and heart failure treatment (HR 1.78, CI 95% 0.99–3.20, *P* = 0.053). In the following nested analyses, GDF-15 and the two echocardiographic measurements all emerged statistically significant when individually added on top of GRACE 2.0. Of the analyzed variables, GDF-15 improved discrimination on top of GRACE 2.0 the most as measured by change of C-index (from 0.77 [CI 95% 0.70–0.85] to 0.81 [CI 95% 0.73–0.89]). The addition of LVEF and GLS to the adjusted model with GRACE 2.0 and GDF-15 only marginally improved the C-index.

**Table 2:** Multivariable Cox regression models of all-cause death at three years follow-up.

Model	Variables	Original HR (CI 95%)	*P*-value	Shrunk HR (CI 95%)	C-index (CI 95%)	Likelihood ratio test (*P*-value for change in model fit)
1	GRACE 2.0, per tertile increase	1.78 (0.99–3.20)	0.053	1.71 (0.99–2.95)	0.774 (0.698–0.849)	-
2	GRACE 2.0, per tertile increase	1.50 (0.83–2.73)	0.183	1.45 (0.84–2.55)	0.775 (0.695–0.856)	0.003*
	LVEF, per % increase	0.96 (0.94–0.99)	**0.002**	0.96 (0.94–0.99)		
3	GRACE 2.0, per tertile increase	1.50 (0.83–2.74)	0.181	1.45 (0.84–2.51)	0.784 (0.703–0.865))	0.004*
	GLS per % increase	1.12 (1.04–1.21)	**0.004**	1.11 (1.04–1.19)		
4	GRACE 2.0, per tertile increase	1.40 (0.78–2.49)	0.260	1.36 (0.80–2.32)	0.811 (0.734–0.887)	< 0.001*
	GDF-15, per tertile increase	3.00 (1.62–5.57)	**< 0.001**	2.75 (1.56–4.85)		
5	GRACE 2.0, per tertile increase	1.19 (0.66–2.15)	0.563	1.17 (0.68–2.02)	0.814 (0.737–0.891)	0.010†
	LVEF, per % increase	0.96 (0.94–0.99)	**0.007**	0.97 (0.94–0.99)		
	GDF-15, per tertile increase	2.84 (1.52–5.28)	**0.001**	2.59 (1.47–4.56)		
6	GRACE 2.0, per tertile increase	1.23 (0.68–2.21)	0.500	1.20 (0.70–2.07)	0.816 (0.736–0.895)	0.035†
	GLS, per % increase	1.11 (1.02–1.20)	**0.011**	1.10 (1.02–1.18)		
	GDF-15, per tertile increase	2.82 (1.52–5.25)	**0.001**	2.58 (1.46–4.55)		

Each Cox-regression model is adjusted for age, sex and heart failure treatment with C-index reported for the entire model. Events at three years follow-up: *n* = 40.

*CI* Confidence Interval, *GLS* Global Longitudinal Strain, *GRACE* Global Registry of Acute Coronary Events, *GDF-15* Growth Differentiation factor 15, *HR* Hazard Ratio, *LVEF* Left Ventricular Ejection Fraction.

^*^Model 1 as reference.

^†^Model 4 as reference.

Multivariable analyses for all-cause death at the end-of-study are reported in Table 3. In the baseline model, GRACE 2.0 was independently associated with all-cause death (HR 1.83, CI 95% 1.28–2.61, *P* < 0.001). LVEF, GLS and GDF-15, all emerged with statistical significance when analyzed in joint models with GRACE 2.0, providing independent prognostic information. Addition of GDF-15 to GRACE 2.0 improved the C-index (from 0.74 [CI 95% 0.69–0.79] to 0.76 [CI 95% 0.72–0.81]) whereas addition of LVEF or GLS only modestly improved discrimination of patients at risk of death at the end-of-study.

**Table 3:** Multivariable Cox regression models of all-cause death at end-of-study (median 6.4 years follow-up).

Model	Variables	HR (95% CI)	*P*-value	C-index (95% CI)	Likelihood ratio test (*P*-value for change in model fit)
1	GRACE 2.0, per tertile increase	1.83 (1.28–2.61)	**< 0.001**	0.740 (0.690–0.790)	-
2	GRACE 2.0, per tertile increase	1.65 (1.15–2.37)	**0.007**	0.752 (0.701–0.804)	0.001*
	LVEF, per % increase	0.97 (0.96–0.99)	**< 0.001**		
3	GRACE 2.0, per tertile increase	1.68 (1.17–2.41)	**0.005**	0.754 (0.702–0.805)	0.003*
	GLS, per % increase	1.08 (1.03–1.13)	**0.003**		
4	GRACE 2.0, per tertile increase	1.60 (1.12–2.29)	**0.010**	0.764 (0.715–0.813	< 0.001*
	GDF-15, per tertile increase	1.81 (1.32–2.47)	**< 0.001**		
5	GRACE 2.0, per tertile increase	1.46 (1.02–2.10)	**0.040**	0.771 (0.721–0.822)	0.004†
	LVEF, per % increase	0.97 (0.96–0.99)	**0.003**		
	GDF-15, per tertile increase	1.74 (1.27–2.38)	**< 0.001**		
6	GRACE 2.0, per tertile increase	1.49 (1.04–2.14)	**0.030**	0.773 (0.723–0.824)	0.008†
	GLS, per % increase	1.07 (1.02–1.12)	**0.008**		
	GDF-15, per tertile increase	1.75 (1.27–2.40)	**< 0.001**		

Each Cox-regression model is adjusted for age, sex and heart failure treatment with C-index is reported for the entire model.

*CI* Confidence Interval, *GLS* Global Longitudinal Strain, *GRACE* Global Registry of Acute Coronary Events, *GDF-15* Growth Differentiation factor 15, *HR* Hazard Ratio, *LVEF* Left Ventricular Ejection Fraction.

^*^Model 1 as reference.

^†^Model 4 as reference.

A comparison between linear and cubic spline models for the studied variables demonstrated minimal improvements in discrimination from the non-linear terms for GRACE 2.0 and LVEF (Supplemental Table 5). Partial effect plots are depicted in Supplemental Fig. 1.

In the explorative analyses at three years (Table 4), GRACE 2.0 demonstrated a higher time-dependent AUC of all-cause mortality than the echocardiographic metrics (GRACE 2.0: 0.76 [0.67–0.84] vs. LVEF: 0.64 [0.54–0.74], *P* = 0.015 and GRACE 2.0: 0.76 [0.67–0.84] vs. GLS: 0.67 [0.57–0.77], *P* = 0.051) although the difference to GLS did not reach statistical significance (Table 2). This superior discrimination from GRACE 2.0 compared to LVEF was sustained at 6- and 9-years follow-up respectively. GDF-15 demonstrated better mortality discrimination than GRACE 2.0 at three years (time-dependent AUC: GRACE 2.0: 0.76 [0.67–0.84] vs. GDF-15: 0.82 [0.75–0.89], *P* = 0.010) and similar discrimination as GRACE 2.0 at 6- and 9-years follow-up.

**Table 4:** Comparison of time-dependent AUC with right-censoring at 3, 6 and 9 years respectively.

Model	AUC at 3 years (CI 95%)	*P*-value	AUC at 6 years (CI 95%)	*P*-value	AUC at 9 years (CI 95%)	*P*-value
GRACE	0.76 (0.67–0.84)	Ref.	0.74 (0.68–0.8)	Ref.	0.76 (0.69–0.82)	Ref.
GDF-15	0.82 (0.75–0.89)	0.010	0.76 (0.7–0.82)	0.529	0.77 (0.7–0.84)	0.721
GLS	0.67 (0.57–0.77)	0.051	0.67 (0.6–0.74)	0.059	0.68 (0.61–0.75)	0.089
LVEF	0.64 (0.54–0.74)	0.015	0.64 (0.57–0.71)	0.009	0.57 (0.49–0.65)	< 0.001

*AUC* Area Under the Receiver Operating Curve, *CI* Confidence Interval, *GLS* Global Longitudinal Strain, *GRACE* Global Registry of Acute Coronary Events, *GDF-15* Growth Differentiation factor 15, *LVEF*, Left Ventricular Ejection Fraction.

**Fig. 2:**
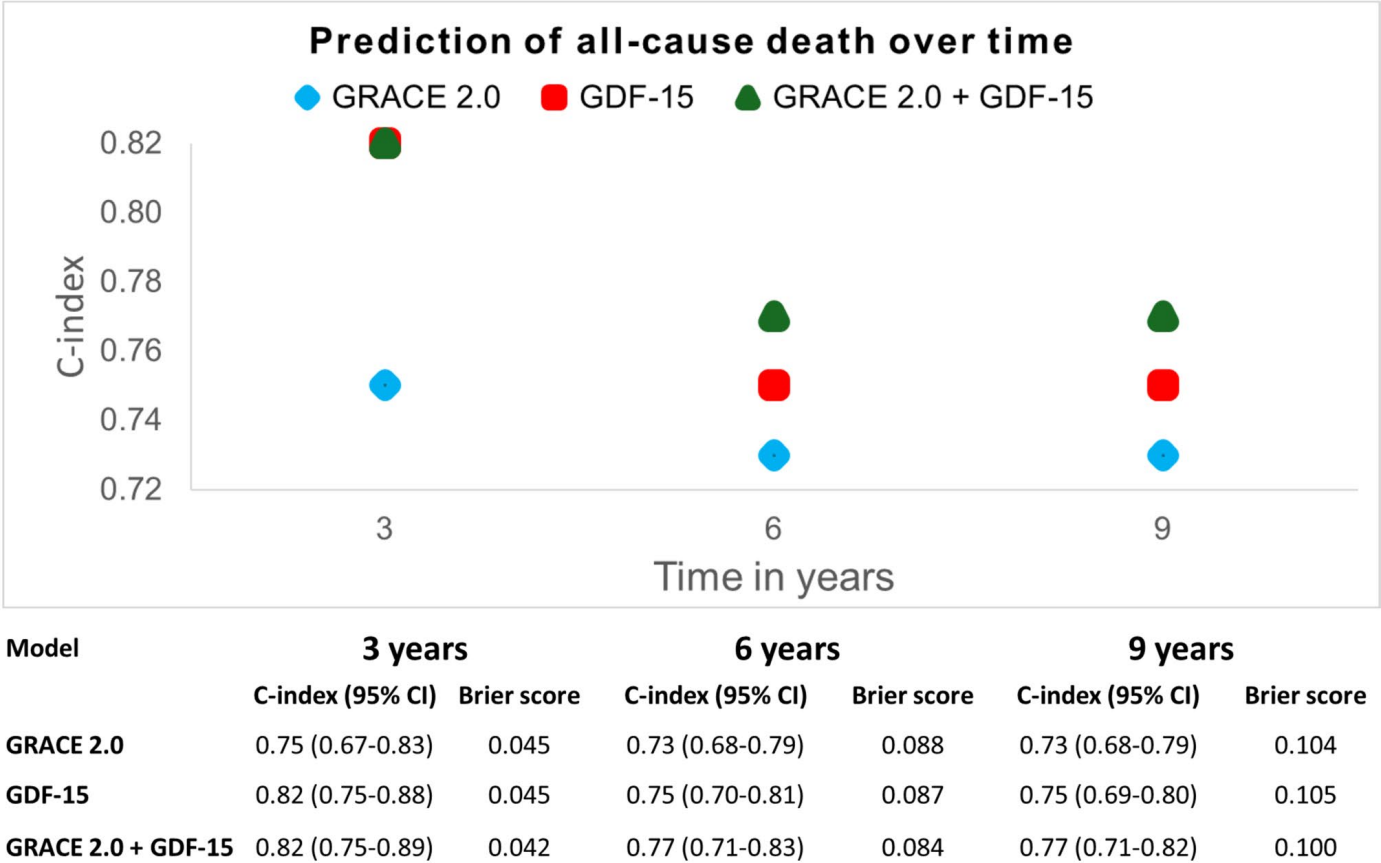
Unadjusted C-index and time-dependent Brier score reported over time for GRACE 2.0, GDF-15 and the combination of both GRACE 2.0 and GDF-15. *CI* Confidence interval, *GRACE* Global Registry of Acute Coronary Events, *GDF-15* Growth Differentiation factor 15.

Figure 2 demonstrates the C-index for GDF-15, GRACE 2.0 and their combination, over time. GDF-15 alone offered superior predictive discrimination of all-cause death at three years as compared to GRACE 2.0 with no additive prognostic value from the combination of the two. At six and nine years, GDF-15 still demonstrated a higher C-index than GRACE 2.0 and there was a small improvement in the C-index by their combined predicted probability. There was a gradual increase in Brier scores over time for all three models, from approximately 0.04 at 3 years to 0.10 at 9 years, reflecting increased uncertainty in long-term risk prediction.

The association between different GDF-15 tertiles and all-cause death is further demonstrated in Supplemental Fig. 2, showing particularly poor prognosis among patients in the third tertile whereas those in the first tertile demonstrated good long-term prognosis. A similar inter-tertile relationship was found for GRACE 2.0 (Supplemental Fig. 3).

## Discussion

Discrimination of patients with high mortality-risk after ACS was substantially improved by the addition of GDF-15 to GRACE 2.0 at three years follow-up and modestly improved at the end-of study (median 6.4 years follow-up). The addition of echocardiographic LVEF or GLS to GRACE 2.0 offered minor improvements in discrimination of patients with high mortality-risk.

Interestingly, at three years follow-up, GDF-15 alone outperformed GRACE 2.0 in prediction of all-cause death with no evidence of any incremental discriminative performance from adding GRACE 2.0 to GDF-15. However, there was benefit in combining GRACE 2.0 and GDF-15 vs. GDF-15 alone for prediction of death beyond three years after ACS.

Early literature on the added prognostic value of LVEF to the original GRACE risk score is conflicting^20^. A Finnish study has recently shown improved prognostication by the addition of LVEF to GRACE in a modern cohort with short-term follow-up of six months^21^, however, intermediate- to long-term follow-up evaluations of incremental benefit from LVEF to GRACE has until now been absent. Similarly, outcome prediction data on the addition of GLS to GRACE is scarce. The closely related metric *global left ventricular myocardial work efficiency*, derived from systolic blood pressure and GLS, have shown promising predictive properties but no earlier studies examining both GLS and GRACE 2.0 in a multivariable model have to our knowledge been published^22^.

Previous analyses from the TOTAL-AMI cohort reported moderate correlation between LVEF and GLS with only a marginal improvement in risk prediction with GLS added to LVEF^5^. It is therefore not surprising to find a similar improvement in the prognostic models from the individual addition of GLS and LVEF to GRACE 2.0.

The study population had predominantly normal or mildly reduced ejection fraction, inferring only a small remaining subgroup with systolic heart failure. GRACE score accounts for Killip-class (i.e., severity of heart failure symptoms) which may blunt the incremental contribution from echocardiographic LV systolic measurements in the multivariable models.

GDF-15 has previously demonstrated a strong improvement in both short- and long-term prognostication to the initial version of GRACE score^23,24^. Despite these findings, GDF-15 has not made it into widespread clinical practice. This can likely be attributed to the poor specificity of GDF-15 upregulation. Although a potent predictor of all-cause death, GDF-15 as an inflammatory and stress-induced cytokine, has been shown to increase in various conditions from cancer to infections and autoimmune disease^25^. Moreover, GDF-15 increases with age and appears not to be a modifiable risk marker^26^. Counter to what may be expected, treatment with SGLT-2-inhibitors over 12 weeks in subjects with reduced ejection fraction was associated with increasing levels of GDF-15 despite evidence of reversed remodeling by reduced LV volumes in a recent publication^27^. Such findings raise questions of correlation and causation regarding GDF-15 and heart disease. Genetic studies by Mendelian randomization have suggested a causative relation to ischemic heart disease but mechanisms are still not fully understood^28^.

Our findings strongly suggest that a modern-day risk prediction score should include GDF-15 given its strong association with mortality. One such alternative score is the above mentioned and recently developed ABC-ACS ischemia score^7^. Risk score calculations can be inconvenient in a busy clinical setting, therefore limiting adoption, and a blood sample with quantification of GDF-15 could therefore be a feasible complementary risk marker to the assessment of GRACE 2^29^. Remaining obstacles to clinical adoption of GDF-15 testing in risk stratification include the absence of prospective validations and limited clinical access to assays.

### Limitations

This study has limitations to address. Given that GDF-15 was assessed with a PEA-method it was not possible to determine a clinically meaningful cut-off for future prospective evaluation. However Peiró and colleagues demonstrated added value of GDF-15 to the original version of GRACE in a Spanish population and suggested an immunoassay derived threshold greater than 1800 ng/L to stratify high risk^6^. This cut off rendered a hazard ratio at 4.09; 95% CI 1.57–10.71) in a model with GRACE, LVEF < 40% and baseline comorbidities.

Our study included a population of selected MI patients as demonstrated by the inclusion and exclusion criteria for enrollment. Additional selection has been driven by exclusion of patients with poor echocardiographic image quality, likely favoring inclusion of more healthy participants. Furthermore, several patients had missing data for calculation of GRACE 2.0 and another few lacked complete biomarker data. However, the final study population appears to be similar to the general ACS population when compared to the large population-based study by Alabas et al.^30^. In our study, included subjects were slightly younger and mildly overrepresented by men yet the prevalence of comorbidities appeared to be similar to the general ACS population.

Timing of the echocardiographic examination may also affect the predictive value of echo-based measures given the occurrence of myocardial stunning days to weeks after ACS^31^. It would have been of interest to also assess myocardial function from a later examination, after potential stunning resolved, yet such data was not available in this real-world observational cohort.

## Conclusion

GDF-15 meaningfully improved discrimination of all-cause death when added on top of GRACE 2.0 whereas LVEF and GLS both provided minor improvements. Furthermore, GDF-15 alone offered better three-year mortality prediction than GRACE 2.0 and provided similar information on mortality risk as GRACE 2.0 beyond three years. These findings support integration of GDF-15 in clinical risk stratification among ACS survivors although further research in larger and more diverse populations is needed.

## Supplementary Information

Below is the link to the electronic supplementary material.

Supplementary Material 1

## Data Availability

Researchers may access the analyzed database at Uppsala Clinical Research Center upon reasonable request and under the provision that the data is accessed onsite and does not leave Uppsala University. This request can be sent to info@ucr.uu.se.
