# Supplementary material for: Growth differentiation factor-15 improves long-term mortality risk prediction beyond the GRACE 2.0 score after acute coronary syndrome
Source: Sci Rep. 2026 Feb 5;16:5241. doi: 10.1038/s41598-026-38905-w (PMC12881424; doi:10.1038/s41598-026-38905-w)

Supplemental data

Inclusion and exclusion criteria for patients admitted with Acute Coronary Syndrome Uppsala University Hospital, Danderyd Hospital or Lund University Hospital.

Inclusion criteria (all have to be met):

- Clinically confirmed acute coronary syndrome (MI or unstable angina)
- Age ≤ 75 years
- Patient included in the SWEDEHEART registry
- Signed informed consent

Exclusion criteria:

- Patient transferred from another clinic
- More than 72 hours from MI diagnosis until planned blood sampling
- Procedural related myocardial infarction (type 4a or type 5)

Supplemental Table 1. Comparison of baseline data between included and excluded patients.

| **Variables** | | **Included**  **N = 751** | | **Excluded**  **N =634** | | **P-value** |
| --- | --- | --- | --- | --- | --- | --- |
| Age, years, mean (SD) | 64.4 (10.0) | | 65.5 (11.3) | | 0.073 | |
| Male sex, n (%) | 583 (77.6) | | 484 (76.3) | | 0.455 | |
| Medical history |  | |  | |  | |
| Hypertension, n (%) | 386 (51.4) | | 286 (45.1) | | 0.011 | |
| Diabetes mellitus, n (%) | 163 (21.7) | | 142 (22.4) | | 0.852 | |
| Heart failure, n (%) | 39 (5.2) | | 48 (7.6) | | 0.079 | |
| History of stroke, n (%) | 49 (6.5) | | 52 (8.2) | | 0.258 | |
| Chronic kidney disease (eGFR < 60mL/min/1.73m^2^), n (%) | 112 (14.9) | | 125 (19.7) | | 0.013 | |
| Prognostic markers |  | |  | |  | |
| GRACE 2.0, probability of death (%), median (IQR)* | 3.5 (2.1-5.7) | | 3.6 (2.1-7.1) | | 0.089 | |
| GDF-15, relative log2-transformed normalized protein expression units), median (IQR)^+^ | 8.51 (8.04-9.00) | | 8.68 (8.19-9.30) | | <0.001 | |

*1165 patients had complete data for GRACE 2.0 computation

^+^1356 patients had data for GDF-15 measurements

Supplemental Table 2. Comparison of baseline data between survivors and non-survivors.

| **Variables** | | **Survivors**  **N = 647** | | **Non-survivors**  **N = 104** | | **P-value** |
| --- | --- | --- | --- | --- | --- | --- |
| Male sex, n (%) | 503 (78.8) | | 76 (73.8) | | 0.293 | |
| Diagnosis of ACS |  | |  | |  | |
| STEMI, n (%) | 307 (47.4) | | 38 (36.5) | | 0.034 | |
| NSTEMI, n (%) | 296 (45.7) | | 54 (51.9) | | 0.255 | |
| GRACE items |  | |  | |  | |
| Age, years, mean (SD) | 63.2 (9.5) | | 72.2 (9.3) | | < 0.001 | |
| Heart rate, mean (SD) | 76 (18.6) | | 84 (22.5) | | < 0.001 | |
| Systolic blood pressure (mmHg), mean (SD) | 149.1 (26.8) | | 146.7 (29.8) | | 0.386 | |
| Creatinine (umol/L), mean (SD) | 84 (33.1) | | 109 (70.3) | | < 0.001 | |
| Cardiac arrest at admission, n (%) | 8 (1.3) | | 1 (1.0) | | 0.847 | |
| ST segment deviation, n (%) | 396 (61.2) | | 55 (52.9) | | 0.073 | |
| Abnormal cardiac Troponin, n (%) | 456 (70.5) | | 82 (78.8) | | 0.079 | |
| Killip class, n (%) |  | |  | | < 0.001* | |
| 1.  2.  3.  4. | 618 (95.5)  24 (3.7)  2 (0.3)  3 (0.5) | | 88 (84.6)  14 (13.5)  2 (1.9)  0 (0.0) | |  | |
| Medical history |  | |  | |  | |
| Hypertension, n (%) | 319 (49.3) | | 57 (64.4) | | 0.004 | |
| Diabetes mellitus, n (%) | 131 (20.2) | | 32 (30.7) | | 0.016 | |
| Heart failure, n (%) | 22 (3.4) | | 17 (16.3) | | < 0.001 | |
| History of stroke, n (%) | 37 (5.7) | | 12 (11.5) | | 0.026 | |
| History of myocardial infarction, n (%) | 105 (16.2) | | 33 (31.7) | | < 0.001 | |
| Chronic kidney disease (eGFR < 60mL/min/1.73m^2^), n (%) | 72 (11.1) | | 40 (38.5) | | < 0.001 | |
| Echocardiographic parameters |  | |  | |  | |
| LVEF, mean (SD) | 54 (10) | | 50 (13) | | < 0.001 | |
| LV GLS, mean (SD) | -15.2 (4.0) | | -13.3 (4.6) | | < 0.001 | |

*Comparison of baseline data between survivors and non-survivors at end-of-study. *Chi^2^-test of the difference between survivors and non-survivors over all Killip class categories.*

Supplemental Table 3. Univariable COX regression with time to all-cause death at three years follow-up as dependent variable

| Model | Variables | HR (CI 95 %) | P-value | C-index |
| --- | --- | --- | --- | --- |
| 1 | GRACE 2.0* | 3.01 (1.84 – 4.91) | < 0.001 | 0.753 |
| 2 | GDF-15* | 3.43 (2.60 – 4.53) | < 0.001 | 0.818 |
| 3 | LVEF | 0.96 (0.93 – 0.98) | < 0.001 | 0.637 |
| 4 | GLS | 1.17 (1.09 – 1.26) | < 0.001 | 0.669 |
| 5 | Age | 1.10 (1.07 – 1.14) | < 0.001 | 0.733 |
| 6 | Male sex | 1.29 (0.64 – 2.58) | 0.477 | 0.524 |
| 7 | Heart failure treatment | 0.64 (0.42 – 0.98) | 0.038 | 0.597 |

**Reported per tertile increase. Events at three years follow-up: n = 40.*

*CI, Confidence Interval; GLS, Global Longitudinal Strain; GRACE, Global Registry of Acute Coronary Events; GDF-15, Growth Differentiation factor 15; HR, Hazard Ratio; LVEF, Left Ventricular Ejection Fraction*

Supplemental Table 4. Univariable COX regression with time to all-cause death at the end-of-study (median 6.4 years) as dependent variable

| Model | Variables | HR (CI 95 %) | P-value | C-index |
| --- | --- | --- | --- | --- |
| 1 | GRACE 2.0* | 2.99 (2.22 – 4.21) | < 0.001 | 0.734 |
| 2 | GDF-15* | 2.70 (2.21 – 3.30) | < 0.001 | 0.743 |
| 3 | LVEF | 0.97 (0.95 – 0.98) | < 0.001 | 0.625 |
| 4 | GLS | 1.13 (1.08 – 1.18) | < 0.001 | 0.651 |
| 5 | Age | 1.10 (1.08 – 1.12) | < 0.001 | 0.713 |
| 6 | Male sex | 1.27 (0.83 – 1.96) | 0.274 | 0.526 |
| 7 | Heart failure treatment | 0.77 (0.59 – 0.99) | 0.048 | 0.552 |

**reported per tertile increase.*

*CI, Confidence Interval; GLS, Global Longitudinal Strain; GRACE, Global Registry of Acute Coronary Events; GDF-15, Growth Differentiation factor 15; HR, Hazard Ratio; LVEF, Left Ventricular Ejection Fraction*

Supplemental Table 5. Performance of linear and spline models evaluated using original C-index, bootstrap-corrected C-index and calibration slope for the full follow-up (median 6.4 years).

| **Model** | **Original**  **C-index** | **Bootstrap-corrected**  **C-index** | **Calibration slope** |
| --- | --- | --- | --- |
| Linear GRACE | 0.735 | 0.728 | 0.94 |
| Spline GRACE | 0.744 | 0.734 | 0.93 |
| Linear LVEF | 0.739 | 0.730 | 0.95 |
| Spline LVEF | 0.749 | 0.738 | 0.93 |
| Linear GLS | 0.741 | 0.736 | 0.97 |
| Spline GLS | 0.744 | 0.733 | 0.95 |
| Linear GDF-15 | 0.764 | 0.758 | 0.97 |
| Spline GDF-15 | 0.768 | 0.756 | 0.93 |

All models include age, sex, and heart failure treatment.

Supplemental Figure 1: Cox models using restricted cubic splines for GDF-15, GRACE score, LVEF, and GLS, each adjusted for age, sex, and heart failure treatment.


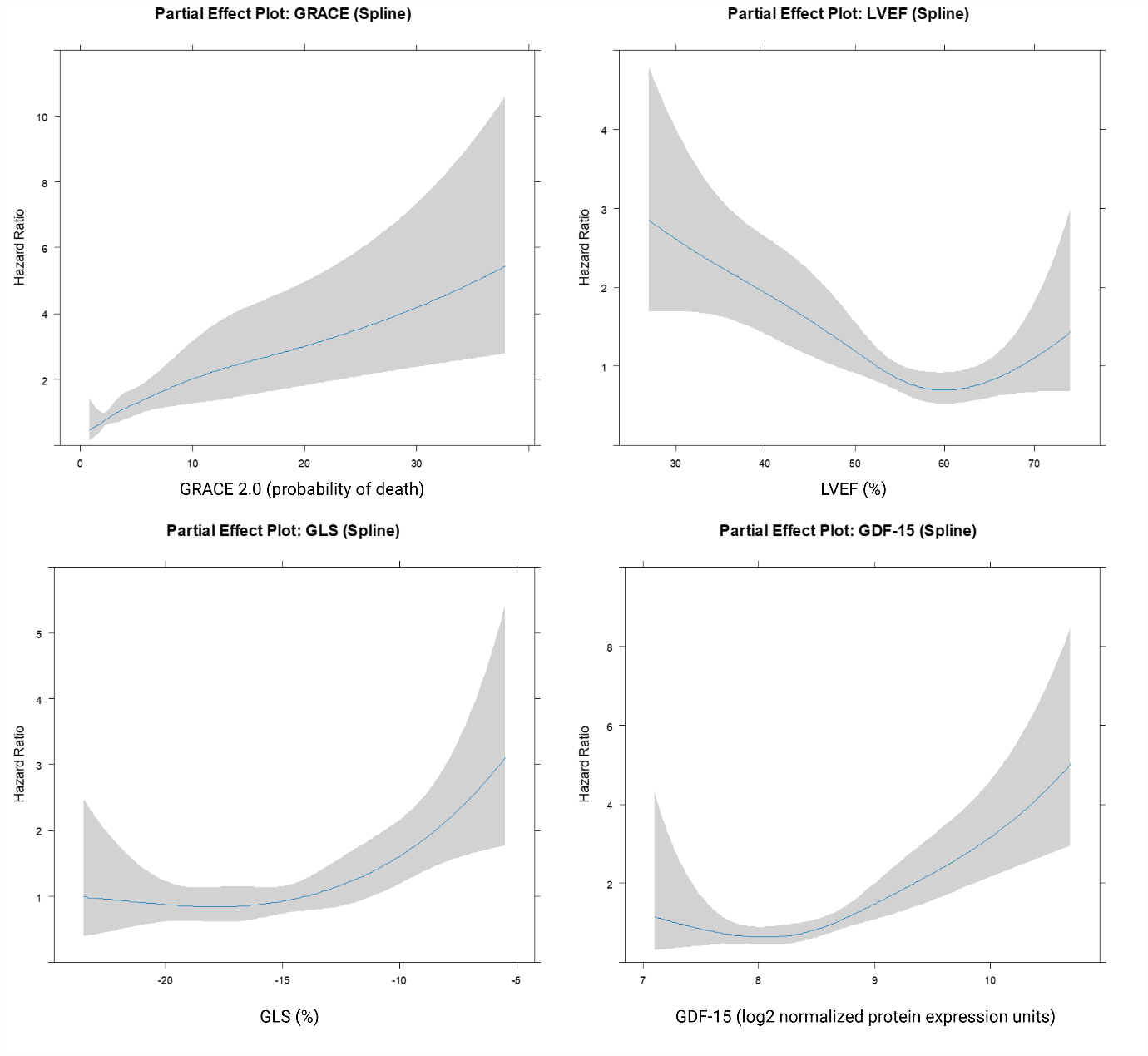


Supplemental Figure 2: Kaplan-Meier curves for assessment of time to all-cause mortality stratified by tertiles of GDF-15


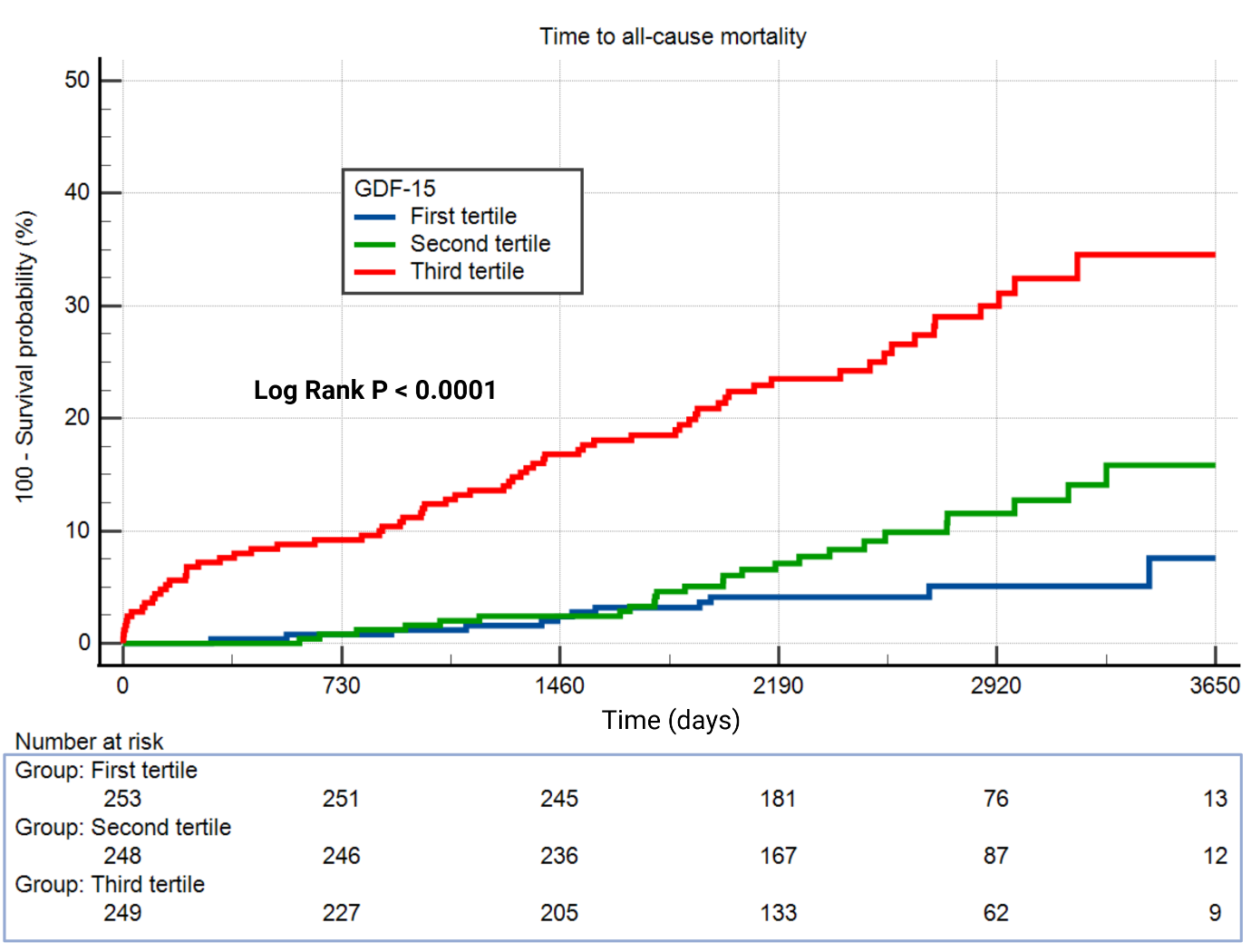


Supplemental Figure 3: Kaplan-Meier curves for assessment of time to all-cause mortality stratified by tertiles of GRACE 2.0


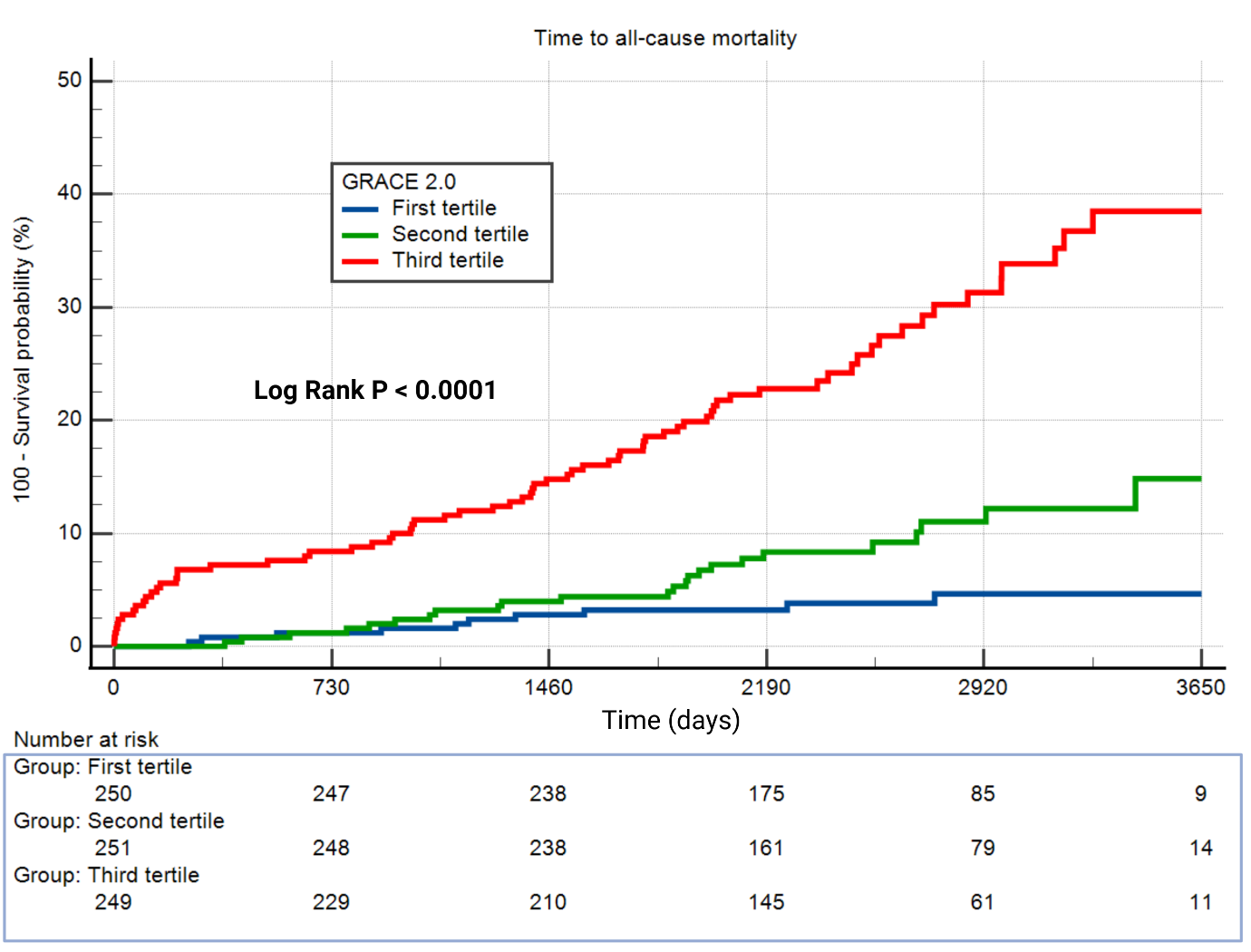

Supplement: Supplementary file 1 — Supplementary Material 1 [file 41598_2026_38905_MOESM1_ESM.docx]
